# Evidence of Neurovascular Un-Coupling in Mild Alzheimer’s Disease through Multimodal EEG-fNIRS and Multivariate Analysis of Resting-State Data

**DOI:** 10.3390/biomedicines9040337

**Published:** 2021-03-26

**Authors:** Antonio M. Chiarelli, David Perpetuini, Pierpaolo Croce, Chiara Filippini, Daniela Cardone, Ludovica Rotunno, Nelson Anzoletti, Michele Zito, Filippo Zappasodi, Arcangelo Merla

**Affiliations:** 1 Department of Neuroscience, Imaging and Clinical Sciences, Institute for Advanced Biomedical Technologies, Faculty of Medicine, University G. D’Annunzio of Chieti-Pescara, Via Luigi Polacchi 13, 66100 Chieti, Italy; david.perpetuini@unich.it (D.P.); pierpaolo.croce@unich.it (P.C.); chiara.filippini@unich.it (C.F.); d.cardone@unich.it (D.C.); f.zappasodi@unich.it (F.Z.); arcangelo.merla@unich.it (A.M.); 2 Department of Medicine and Science of Ageing, Faculty of Medicine, University G. d’Annunzio of Chieti-Pescara, Via Dei Vestini 31, 66100 Chieti, Italy; ludorotunno@gmail.com (L.R.); nelson.anzoletti@gmail.com (N.A.); m.zito@dmsi.unich.it (M.Z.)

**Keywords:** Alzheimer’s disease (AD), electroencephalography (EEG), functional near-infrared spectroscopy (fNIRS), multimodal neuroimaging, neurovascular coupling (NC), multivariate analysis

## Abstract

Alzheimer’s disease (AD) is associated with modifications in cerebral blood perfusion and autoregulation. Hence, neurovascular coupling (NC) alteration could become a biomarker of the disease. NC might be assessed in clinical settings through multimodal electroencephalography (EEG) and functional near-infrared spectroscopy (fNIRS). Multimodal EEG-fNIRS was recorded at rest in an ambulatory setting to assess NC and to evaluate the sensitivity and specificity of the methodology to AD. Global NC was evaluated with a general linear model (GLM) framework by regressing whole-head EEG power envelopes in three frequency bands (theta, alpha and beta) with average fNIRS oxy- and deoxy-hemoglobin concentration changes in the frontal and prefrontal cortices. NC was lower in AD compared to healthy controls (HC) with significant differences in the linkage of theta and alpha bands with oxy- and deoxy-hemoglobin, respectively (*p* = 0.028 and *p* = 0.020). Importantly, standalone EEG and fNIRS metrics did not highlight differences between AD and HC. Furthermore, a multivariate data-driven analysis of NC between the three frequency bands and the two hemoglobin species delivered a cross-validated classification performance of AD and HC with an Area Under the Curve, AUC = 0.905 (*p* = 2.17 × 10^−5^). The findings demonstrate that EEG-fNIRS may indeed represent a powerful ecological tool for clinical evaluation of NC and early identification of AD.

## 1. Introduction

Alzheimer’s disease (AD) is a form of dementia that usually begins with slight memory failures and slowly progresses into evident cognitive impairment [1,2,3]. AD is the cause of about 70% of cases of elderly dementia [4]. The underlying mechanisms of the clinical symptoms of AD are poorly understood [5]. However, AD is known to be associated with extracellular amyloid beta deposits [6,7], tau protein abnormalities [8,9] and neuronal loss [10,11]. Moreover, AD has been recently linked to early neurovascular dysfunction, which clearly contributes to disease pathogenesis [12,13,14]. In this perspective, probing functional hyperemia, i.e., neurovascular coupling (NC), could provide relevant diagnostic and prognostic neuromarkers of the disease [15,16,17].

Neurovascular coupling is a physiological mechanism that refers to the causal relationship between local neural activity and subsequent increases in cerebral blood flow (CBF) through cerebrovascular reactivity (CVR) [18,19,20,21]. NC ensures a rapid augmentation in CBF and oxygen delivery to activated brain structures. The increase in oxygenated blood delivery overcompensates the rise in the local cerebral metabolic rate of oxygen (CMRO_2_), effectively, and paradoxically, reducing the regional oxygen extraction fraction (OEF) [22]. This decrease in OEF can be probed by functional neuroimaging technologies by assessing the local increase in oxy-hemoglobin (HbO) coupled with a regional decrease in deoxy-hemoglobin (HbR). The NC mechanism is commonly exploited in functional magnetic resonance imaging (fMRI) to infer brain activity from the hemodynamic signal [23,24]. In fMRI, functional hyperemia induces an increase in signal intensity driven by a reduction of the paramagnetic HbR and it is generally referred to as the blood oxygen level-dependent (BOLD) effect [25].

Functional near-infrared spectroscopy (fNIRS) is an ecological alternative to fMRI that has become a useful tool for brain activity monitoring due to its portability, low invasiveness and limited cost [26,27]. fNIRS is a scalp-based optical technology that uses light injection and detection points to probe brain tissues. fNIRS relies on multiwavelength differential measurements of the backscattered light which is sensitive to changes in the concentration of the main oscillating chromophores in the near-infrared (NIR) spectral range (wavelengths between 650 and 900 nm), namely HbO and HbR. This characteristic, together with the highly diffusive properties and low absorption of biological tissues in NIR, allows us to probe the cortex and to measure changes in the concentration of HbO and HbR directly from the scalp. In recent years, fNIRS has become a common brain imaging method for studying different populations and experimental conditions [28,29,30,31,32,33,34].

Functional neuroimaging technologies, such as fMRI and fNIRS, provide a powerful tool to study brain activity. However, since neuronal activation is inferred indirectly from a hemodynamic signal, they require a priori assumptions about its temporal characteristics [24]. In general, neuronal activity is assumed to perfectly match the stimulation which, for this reason, is performed through temporally structured tasks [35]. Moreover, the hemodynamic shape in response to a short stimulation (i.e., the hemodynamic response function, HRF) is assumed to be known, or only slight modifications are allowed, effectively providing stringent constraints on the shape of NC [36]. An alternative approach is to use functional neuroimaging technologies to directly assess the hemodynamic response function and the NC [37,38,39]. In this case, a priori assumptions about the duration of the underlying neuronal activity are also required; this implies employing temporally paced stimulations. Indeed, multimodal approaches are ideal to probe brain activity and NC with fewer a priori assumptions. Technologies that measure electrical brain activity, such as electroencephalography (EEG) [40], and magnetoencephalography (MEG) [41], can be used together with functional neuroimaging technologies, such as fMRI and fNIRS, to concurrently measure electrical and hemodynamic brain fluctuations [42,43,44] and deliver NC estimates in the absence of structured tasks, for example, during a resting state [45].

To this end, combining fNIRS with synchronous EEG is gaining increasing interest in research and clinical settings [46]. Because of the absence of electro-optical interference with these two scalp-located technologies, it is simple to integrate fNIRS with EEG. The simplicity of the hardware integration also encourages the development of unified analytical frameworks [19,47,48]. EEG is a well-established technique in neurological and neuroimaging settings suitable for capturing the temporal dynamics of brain electrical activity through measurements of voltages generated by macroscopic currents within neuronal ensembles [49]. EEG systems are widely used for research and clinical purposes to monitor and diagnose brain function and dysfunction [50].

EEG-fNIRS multimodal systems have already been employed to identify functional modifications associated with AD. For example, Li et al. recently evaluated EEG-based dynamic cortical connectivity alterations associated with AD and used fNIRS-based spatial constraints as priors for EEG source localization [51]. Moreover, Cicalese and colleagues [52] used a hybrid EEG-fNIRS system to perform a data-driven identification of AD based on EEG and fNIRS features evaluated on recordings performed during a random digit encoding retrieval task. They obtained sufficiently good classification performances (around 80% in a four-class classification) which were superior to those obtained employing standalone modalities. Notably, while they did combine EEG and fNIRS features in a multivariate data-driven analysis, they did not evaluate cross-modality metrics related to NC. In fact, although EEG-fNIRS systems have been employed to extract cross-modality metrics of NC in different clinical populations, such as stroke patients [53], neonates [54], patients with acute brain injury [55] and in the assessment of physiological aging [56], to our knowledge, such multimodal methodology was not applied for the evaluation of NC and its modification in AD.

In this study, a comparison of resting-state EEG-fNIRS NC between AD and healthy controls (HC) was performed. The extent of NC was assessed by correlating whole-head EEG power envelopes in three frequency bands (theta (θ), alpha (α) and beta (β)) with changes in HbO and HbR, assessed through fNIRS in the frontal and prefrontal cortices (six NC metrics). The coupling between electrical and hemodynamic brain activity was evaluated employing a general linear model (GLM) [57,58,59,60]. The GLM is a standard approach to infer brain activity from functional neuroimaging technologies such as fNIRS and fMRI, and it is a generalization of univariate regression. In this context, GLM allowed us to concurrently evaluate the coupling between multiple EEG frequency bands and hemoglobin species. Finally, a multivariate data-driven (i.e., machine learning) analysis of the six global NC metrics was performed to demonstrate the statistical robustness of the findings and to deliver an approach that could support AD diagnosis in clinical settings.

## 2. Materials and Methods

### 2.1. Participants

Thirty-five participants were recruited for the study. The population was composed of 17 AD patients and 18 healthy controls (HC, refer to Table 1 for demographic information). The AD patients had a diagnosis of mild probable Alzheimer’s disease, as defined by the Diagnostic and Statistical Manual of Mental Disorders, 5th edition (DSM-5). Patients with moderate cognitive impairment (Mini Mental State Examination, MMSE, < 20/30) [61], vascular dementia, behavioral or psychiatric disorders, brain lesions or with a history of stroke or traumatic brain injury were excluded.

### 2.2. Experimental Design

Resting-state recordings of 5 min in duration were performed after the administration of the MMSE. The patients sat comfortably on a chair in front of a doctor, and they were asked to stay as still as possible with their eyes closed and to not think anything specific during the recordings.

### 2.3. EEG Instrumentation and Measurements

**Figure 1:**
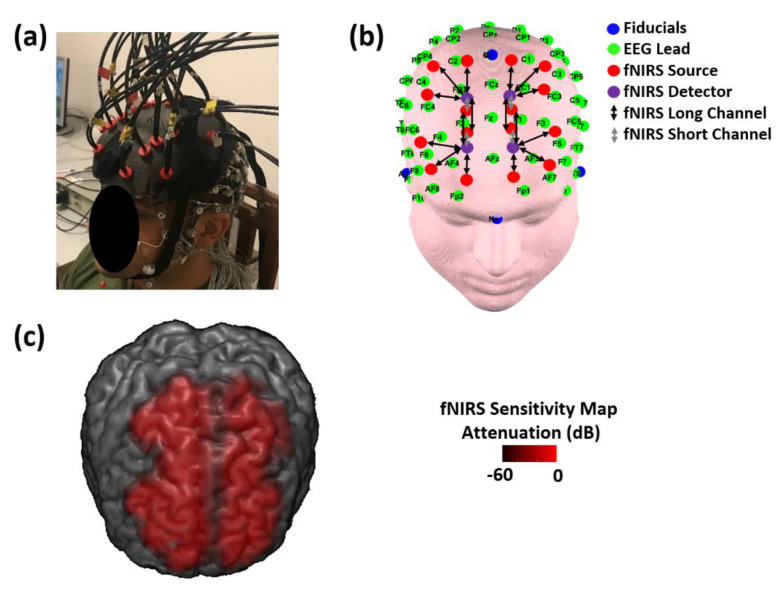
(**a**) Electroencephalography and functional near-infrared spectroscopy (EEG-fNIRS) probes located on a participant’s head. The fNIRS fiber heads reached the scalp using the spaces between the electrodes of the EEG cap. (**b**) EEG-fNIRS probes overlaid on a template head. The high-density (128-channel) EEG layout was arranged in agreement with the 10/20 system and the fNIRS probes were positioned with reference to the EEG sensors. (**c**) Channels’ average sensitivity map of the fNIRS measurements. The sensitivity map was computed using the finite element method to solve the diffusion equation [67] and it is displayed up to an attenuation of 60 dB from its maximum value.

A high-density, 128-channel, full-head EEG system (Electrical Geodesic Inc., Eugene, OR, USA, EEG System Net 300, Figure 1a) was used to record brain electrical activity. Skin/electrode impedance was measured before recordings and kept below 50 kΩ. Notably, 50 kΩ is considered an impedance within the optimal range for the EEG system employed. Although a sensor-to-scalp impedance below 5 kΩ is generally required for EEG recordings, the HydroCel Geodesics Sensor Net provides excellent signals with impedances up to 50–100 kΩ thanks to the high input impedance amplifiers [62]. EEG data were sampled at 250 Hz.

### 2.4. FNIRS Instrumentation and Measurements

A frequency domain near-infrared spectroscopy (NIRS) system (Imagent, ISS Inc., Champaign, IL, USA) was used to perform the optical measurements. The system was made of 32 laser diodes sources, 16 emitting at 690 nm wavelength and 16 emitting at 830 nm wavelength, and 4 photomultiplier tube (PMT) detectors. The lasers were modulated at 110 MHz, whereas the PMTs were modulated at 110 MHz and 5 KHz for heterodyne detection of the modulated light. A time multiplexing scheme was used on the sources to avoid their crosstalk. The measurement sampling rate (accounting for source time-multiplexing) was set at 10.42 Hz. The light intensity delivered to the scalp was below 4 mW/cm^2^, within the ANSI standard safe limits. NIR light was carried to the scalp using optic fibers with a 400 μm core (2 fibers reached each light injection location delivering light from one laser diode emitting at 830 nm wavelength and one laser diode emitting at 690 nm wavelength) and from the scalp back to the PMTs using fiber bundles with a diameter of 3 mm.

The fibers were located on the frontal and prefrontal areas and were held in place with a home-made optical patch located on top of the high-density EEG cap (Figure 1a). The fiber heads reached the scalp using the space among the electrodes of the EEG cap, that were also employed for positioning of the optical array with reference to the 10/20 system [63] (Figure 1b). Notably, the need to develop a home-made optical patch was driven by the necessity to locate fNIRS optodes in between EEG electrodes that were arranged in a high-density configuration and that were around 1 cm thick. These mechanical constraints led us to develop a home-made patch that was able to hold the optodes on top of the EEG cap while allowing them to reach the scalp surface for a correct optode-to-scalp coupling using the small space available among EEG electrodes. The optical array allowed us to perform fNIRS measurements from 16 channels at source–detector distances of 35 mm (long separation channels), probing the brain cortex (Figure 1b,c), and from 4 short separation channels at a 15 mm interoptode distance (Figure 1b). These short separation channels were used to selectively measure hemoglobin concentration changes in the scalp and to correct for their contamination in the long separation channels [64,65].

Slightly longer interoptode distances were employed compared to standard practices for both long and short separation channels (35 mm vs. ~30 mm and 15 mm vs. ~10 mm) [64]. These interoptode distances were preferred because of the age range and the disease of the population examined. Brain atrophy associated with aging and AD tends to increase the distance of the cortical surface from the scalp. Moreover, only 4 short separation channels were used because of the limited number of optodes available. However, because of the not exceedingly large area covered by the optical array, the average distance between the centroid of the closest short separation channel and the centroid of each long separation channel was 18 mm, which is considered a good compromise [66].

### 2.5. EEG Pre-Processing

**Figure 2:**
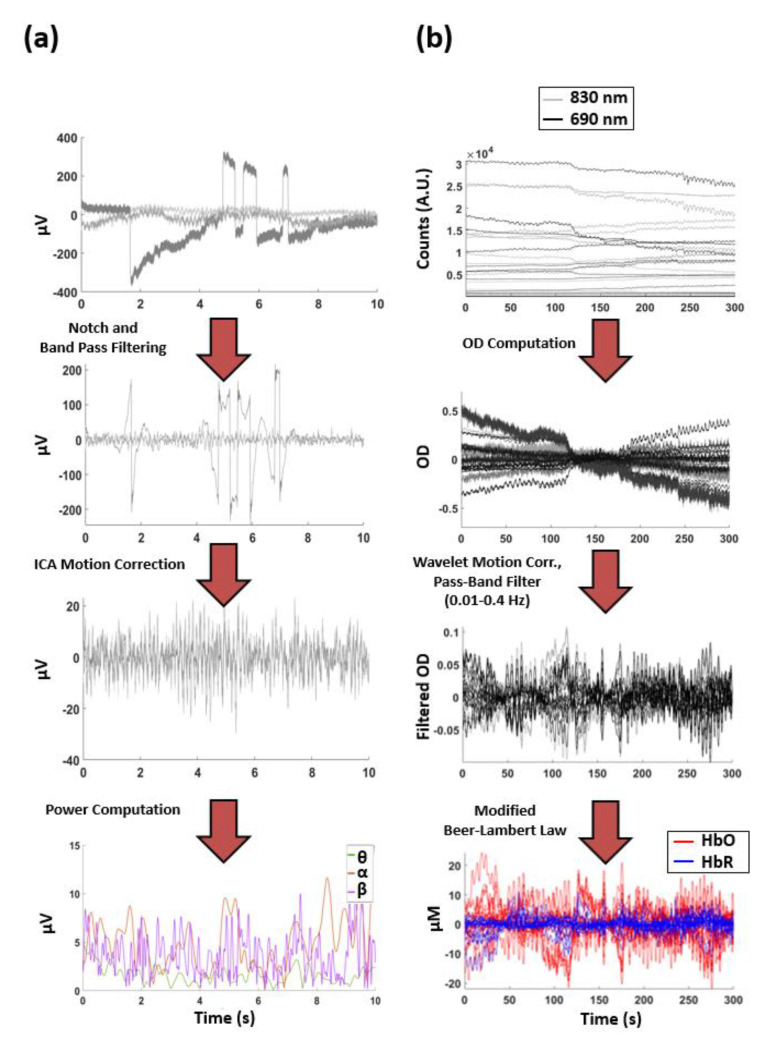
(**a**) Pre-processing of the EEG involving band-pass filtering, independent component analysis (ICA) motion correction and power envelope computation. The end results of the EEG pre-processing were artifact-corrected EEG power envelopes for the 128 channels in the three frequency bands of interest (theta, alpha and beta). (**b**) Pre-processing of the fNIRS involving Optical Densities (OD) computation, wavelet-based motion correction and oxy-hemoglobin (HbO) and deoxy-hemoglobin (HbR) computation. The end results of the fNIRS pre-processing were artifact-corrected HbO and HbR changes in both the long and the short separation channels.

The flowchart of EEG pre-processing is reported in Figure 2a. EEG data were visually inspected to reject saturated or corrupted epochs. Data were then band-pass filtered between 1 and 80 Hz and notch-filtered at 50 Hz (zero-lag 2nd order Butterworth digital filters). Furthermore, a semiautomatic procedure based on independent component analysis (ICA) was applied to identify and to remove cardiac and ocular artifacts, as well as signals coming from muscles [68,69]. The filtered and artifact-corrected EEG signals were decomposed in three frequency bands of interest (i.e., theta: 3.5–8.2 Hz, alpha: 7.4–13 Hz and beta: 13–30 Hz) and the power temporal envelopes were computed as the absolute values of their Hilbert transform.

### 2.6. FNIRS Pre-Processing

The flowchart of fNIRS pre-processing is reported in Figure 2b. The raw continuous-wave components of the acquired signals were converted into optical densities (ODs) according to the equation:

(1) $$\mathrm{OD}\;=\;-\ln\left(\frac{I\left(t\right)}{I_{m}}\right)$$

where I(t) is the time dependence of the recorded signal intensity and I_m_ is its average value during rest. Furthermore, motion artifacts were corrected by applying a wavelet-based procedure [70] and the ODs were band-pass filtered with a zero-lag, 4th order Butterworth digital filter with cut-off frequencies of 0.01 Hz and 0.4 Hz. These motion correction and filtering approaches allowed us to highlight the hemodynamic components of the acquired optical signal. Notably, the recordings were performed standing still and at rest and they were poorly contaminated by motion-related signals. Thus, the use of a motion correction algorithm that does not distort the data in the presence of a low level of motion noise was preferred (kurtosis-based wavelet filtering) [70]. After visual inspection of the filtered and motion-corrected ODs, changes in the concentration of HbO and HbR were derived for the rest period from each channel based on the modified Lambert–Beer law [71]:

(2) $$\left[\begin{array}{c} \mathrm{HbO}\\ \mathrm{HbR} \end{array}\right]\;=\;\frac{1}{\rho }{\left[\begin{array}{cc} \varepsilon_{\mathrm{HbO}-830\mathrm{nm}}\cdot {\mathrm{DPF}}_{830\mathrm{nm}}\; & \varepsilon_{\mathrm{HbR}-830\mathrm{nm}}\cdot {\mathrm{DPF}}_{830\mathrm{nm}}\\ \varepsilon_{\mathrm{HbO}-690\mathrm{nm}}\cdot {\mathrm{DPF}}_{690\mathrm{nm}}\; & \varepsilon_{\mathrm{HbR}-830\mathrm{nm}}\cdot {\mathrm{DPF}}_{690\mathrm{nm}} \end{array}\right]}^{-1}\times \left[\begin{array}{c} {\mathrm{OD}}_{830\mathrm{nm}}\\ {\mathrm{OD}}_{690\mathrm{nm}} \end{array}\right]$$

where ρ is the interoptode distance (either 35 mm or 15 mm), ε is the extinction coefficient for each chromophore and wavelength and DPF is the differential pathlength factor for each wavelength. The extinction coefficients were extracted from Zijlstra et al. [72], whereas the DPFs [73], accounting not only for wavelength used but also for subject’s age, were extracted from Scholkmann and Wolf [74].

### 2.7. Neurovascular Coupling Estimation

**Figure 3:**
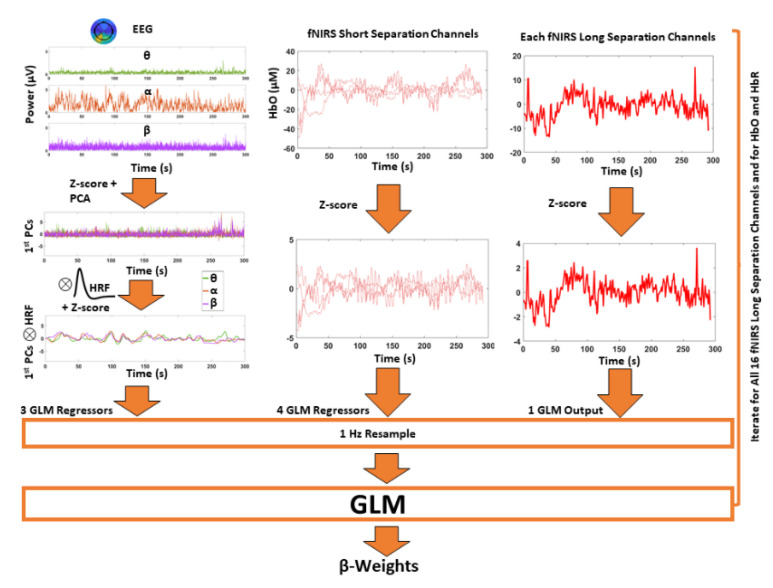
Algorithm for neurovascular coupling (NC) estimation from EEG-fNIRS recordings using a general linear model (GLM) framework. The independent variables of the GLM were the expected hemodynamic responses (z-scored, 1 Hz resampled) obtained from the convolution of the global EEG power envelopes with the canonical hemodynamic response function (HRF). The global EEG power envelopes were obtained by selecting the 1st principal components (PCs) of bandwise principal component analyses (PCAs). Control for scalp contamination was obtained by considering hemoglobin oscillations in the short separation fNIRS channels as additional independent variables (z-scored, 1 Hz resampled). The dependent variable was either HbO or HbR signal in each long separation fNIRS channel (z-scored, 1 Hz resampled). The analysis provided standardized β-weights depicting the strength of association (NC) between bandwise global EEG power envelopes and HbO and HbR in each long separation channel considered.

The algorithmic approach for NC estimation is reported in Figure 3. For each subject and frequency band, EEG power timecourses were normalized (z-scored) and fed to a temporal principal component analysis (PCA). This analysis provided temporally independent timecourses (principal components, PCs) that were common to the different EEG channels within each band. The first PC, i.e., the component that explained the largest amount of signal variance, was retained for each frequency band. This analysis effectively provided a global EEG signal for each frequency band which was not obtained through simple arithmetic averaging among channels but through weighted averaging that accounted for temporal correlation among the different channels. As the EEG signal is highly correlated among channels due to volume conduction effects, this approach is intrinsically robust to non-homogenous noise across channels.

The first PCs of the three EEG frequency bands were convolved with a canonical HRF [24] to obtain a model of the expected hemoglobin concentration changes following resting-state electrical brain activity. These three global signals were resampled (at 1 Hz) and normalized (z-score) and then they were considered as independent variables of the GLM. The normalized (z-score) and resampled (at 1 Hz) HbO and HbR changes in the long separation fNIRS channels were employed as dependent variables. In order to regress out the scalp contamination in the long separation channels, the normalized hemoglobin changes in the four short separation fNIRS channels were also considered as independent variables. The NC estimate was encoded in the β-weight, that, given the normalization of the signals prior to the GLM, delivered a standardized regression coefficient of each EEG power envelope association with the HbO and HbR changes for each of the long separation fNIRS channels. Notice that, whereas the GLM has already been used in EEG-fMRI studies [75,76], it is novel in the EEG-fNIRS investigation of brain activity and NC.

In order to prove the utility of the EEG-fNIRS multimodal approach and GLM for studying the NC in AD, unimodal electrical and hemodynamic brain activities and their changes with AD were investigated as well. In particular, the average EEG and fNIRS powers were evaluated by measuring the mean value of the EEG power in the theta, alpha and beta bands and the standard deviation in the HbO and HbR fNIRS timecourses.

### 2.8. Statistical Inference and Multivariate Data-Driven Analysis

The global NC of the three EEG frequency bands with HbO and HbR (6 metrics of NC for each subject) were evaluated by computing the average of the GLM β-weights among the fNIRS long separation channels.

The global electrical and hemodynamic brain activities were estimated by computing the channels’ average EEG power in the theta, alpha and beta bands (3 metrics of electrical brain activity for each subject) and the channels’ average fNIRS power (i.e., signal standard deviation) for HbO and HbR timecourses (2 metrics of hemodynamic brain activity for each subject).

In order to test the statistical significance of NC in AD and HC and test between-groups differences, *t*-tests were performed [77].

Once univariate statistical significance was explored, in order to further corroborate the results and to explore the clinical significance of the findings, a multivariate data-driven analysis was implemented [78]. A multivariate regression analysis was performed in the attempt to classify each subject as AD or HC based on EEG, fNIRS and NC metrics. Since the classification was known, such an approach represented a classical problem of supervised learning in a machine learning framework [79]. Although several machine learning approaches could be suitable for such a purpose, given the limited number of independent features available and the exploratory nature of the implemented approach, the procedural complexity was limited to the use of a simple linear regressor followed by an output threshold selection. Moreover, the use of a linear regression avoided investigation of the model hyperparameter space, which is compulsory if regularization approaches or non-linear regressors are used (e.g., choosing the regularization parameter or the non-linearity extent). The closed solution and the small number of effective degrees of freedom of the linear regressor (equal to the number of the independent features) greatly reduced the chance of overfitting [80].

In order to build and test the multivariate linear regression, a value of “1” and “0” was attributed to AD and HC, respectively, and the model was trained on all subjects except one and tested on the remaining subject in an iterative manner (i.e., using the leave-one-out cross-validation (CV) scheme) [81,82]. The classification outcome was assessed through a receiver operating characteristic (ROC) analysis of the out-of-training-sample outputs of the iteratively trained regressor which, given the labeling value associated with AD and HC, tended to provide larger values for the AD group. The framework was implemented multiple times to estimate the capability of the procedure to separate AD from HC using the 6 global EEG-fNIRS NCs, the 3 global EEG powers in the theta, alpha and beta bands and the 2 global fNIRS powers for the HbO and HbR timecourses.

In order to investigate the effect of the EEG channel distribution on the global characteristics of the NC metrics, the exact same multivariate data-driven analysis using NC metrics was performed again, but selectively evaluating the EEG signals acquired with half of the available electrodes, all located on the frontal and prefrontal brain areas, while excluding the occipital ones.

Finally, in order to evaluate the performance of the proposed approach as a function of practical variables of interest, such as EEG-fNIRS recording time and number of EEG channels employed, the multivariate data-driven analysis was conducted multiple times by changing either the temporal duration of the signals used (from 15 s to 300 s with a 15 s step) or the number of channels employed (from 4 to 128 with a 4-channel step). The different time windows were selected at the center of the recordings for each subject, whereas the subsample of channels was randomly selected from the 128 channels covering the whole head. When the 300 s window or the 128 channels were evaluated, the analyses were equivalent to the original one where all the signals recorded were employed for NC evaluation and AD vs. HC classification.

## 3. Results

Table 1 reports demographic information for AD and HC. Notably, AD and HC were matched for gender, age and education. A lower MMSE score was identified in AD (t = 5.51, df = 33, *p* = 4.05 × 10^−6^).

The first PCs of the EEG power envelopes explained an average 64% (SD = 13%), 68% (SD = 14%) and 69% (SD = 12%) of the variance in the theta, alpha and beta bands, respectively.

**Figure 4:**
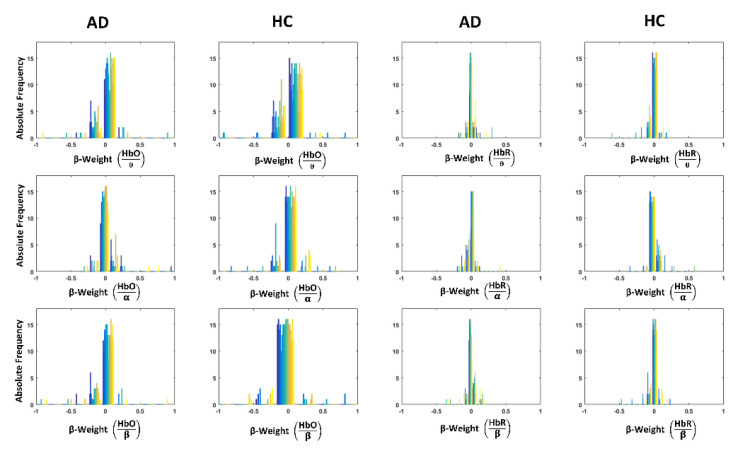
Distributions of the GLM standardized β-weights for the fNIRS long separation channels depicting the linkage of the 1st PCs of the three EEG power envelopes in theta (θ), alpha (α) and beta (β) bands with HbO and HbR changes in AD and HC. The different colors represent different subjects.

Figure 4 shows the distributions of the GLM standardized β-weights for the long separation fNIRS channels in the different subjects (reported with different colors) for the three EEG frequency bands and HbO and HbR in AD and HC.

**Figure 5:**
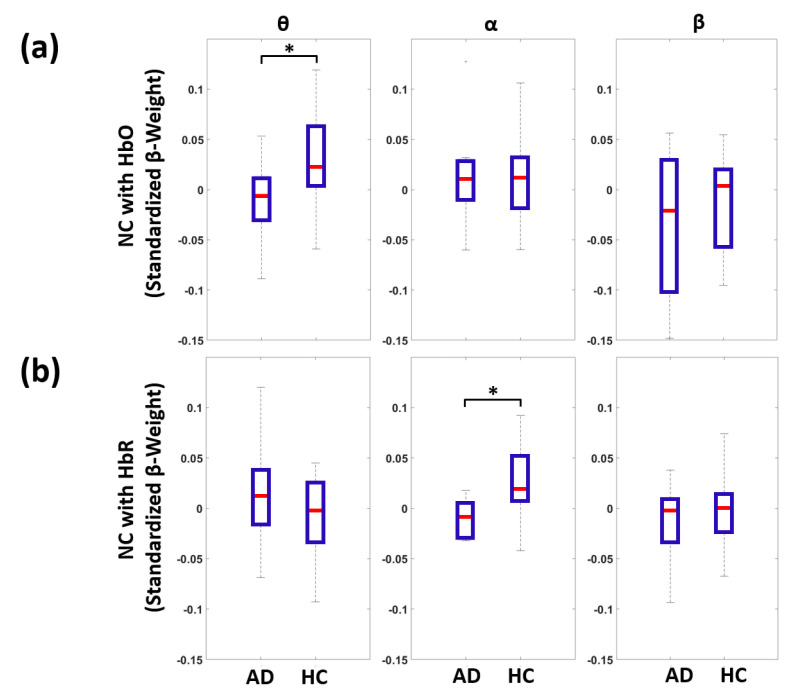
Boxplots showing the average standardized β-weights among the long separation fNIRS channels (global NCs) for AD and HC. (**a**) Global NC of theta, alpha and beta bands with HbO. (**b**) Global NC of theta, alpha and beta bands with HbR (* *p* < 0.05).

Figure 5 reports the boxplots of the average GLM standardized β-weights (global NCs) computed among the long separation fNIRS channels. Figure 5a reports the NC of theta, alpha and beta bands with HbO for AD and HC, whereas Figure 5b reports the NC of theta, alpha and beta bands with HbR for AD and HC.

A statistically significant NC was obtained for HC in the coupling between theta band and HbO (t = 2.12, df = 17, *p* = 0.049) and in the coupling between the alpha band and HbR (t = 2.24, df = 17, *p* = 0.039). This link was lost in AD (*p* > 0.05). In fact, a statistically significant decrease in global NC in AD with respect to HC was obtained for the coupling between the theta band and HbO (t = −2.30, df = 33, *p* = 0.028) and for the alpha band and HbR (t = −2.44, df = 33, *p* = 0.020). Notably, no NC was statistically above zero and no significant increase in global NC was obtained for AD with respect to HC.

**Figure 6:**
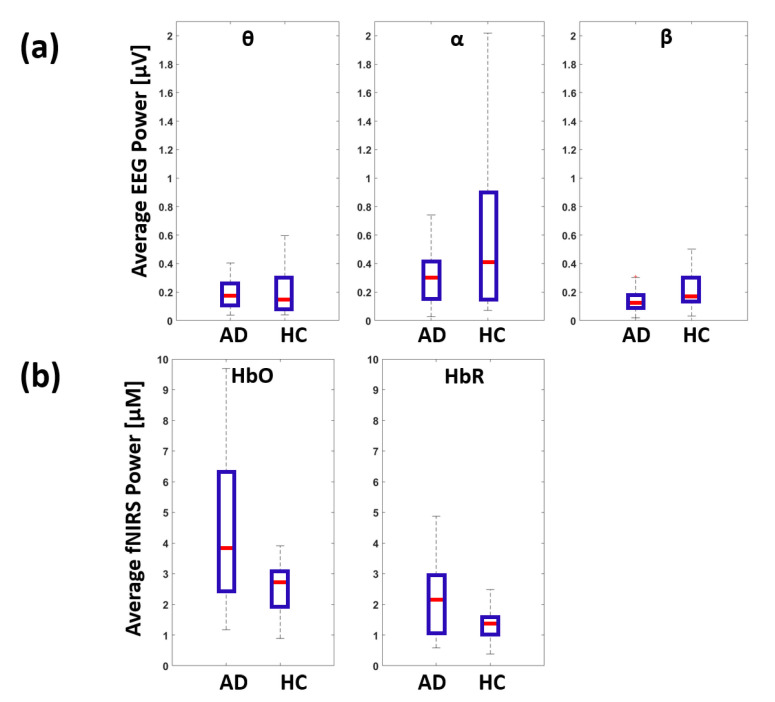
Boxplots showing the EEG and fNIRS average power for AD and HC. (**a**) Global EEG power in theta (θ), alpha (α) and beta (β) bands. (**b**) Global fNIRS power in HbO and HbR timecourses.

For comparison, Figure 6 reports the boxplots showing global metrics of electrical and hemodynamic brain activity evaluated through standalone EEG and fNIRS. Figure 6a reports the global EEG power in theta, alpha and beta bands for AD and HC. Figure 6b reports the global HbO and HbR power for AD and HC. No statistically significant differences were found between AD and HC for the metrics evaluated through unimodal recordings.

**Figure 7:**
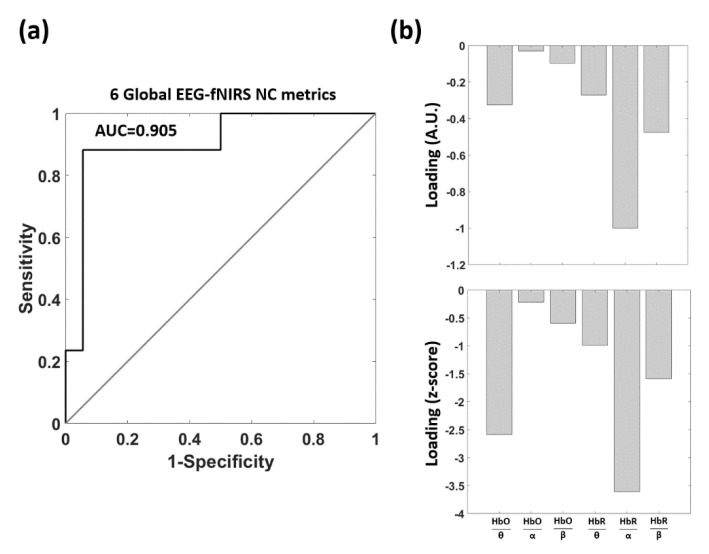
Cross-validation (CV) classification outcome of the multivariate data-driven analysis predicting AD and HC from the 6 NC metrics. (**a**) Receiver operating characteristic (ROC) analysis. (**b**) Upper panel: model loadings; lower panel: z-scores obtained as loadings normalized by a bootstrap estimate of their standard deviation.

Figure 7 reports the outcome of the multivariate data-driven analysis trying to predict AD and HC from the six NC metrics. Importantly, the results were obtained using a leave-one-out CV, depicting the generalization performance of the approach. Figure 7a reports the outcome of the ROC analysis where an area under the curve (AUC) of 0.905 (z = 4.090, *p* = 2.17 × 10^−5^) was obtained. Table 2 reports the confusion matrix that was computed by selecting the threshold of the outcome of the multivariate linear regression that provided a sensitivity of 88.2% and a specificity of 94.5%. Figure 7b shows the CV loadings of the multivariate regression analysis, showing how the different features were concurrently weighted to deliver the classification. Since in the classification procedure AD had a value of “1”, whereas HC had a value of “0”, a positive load depicts a larger NC for AD whereas a negative load represents a smaller NC for AD. Notice that, whereas the upper panel of Figure 7b reports the regression loadings, the lower panel of Figure 7b reports the loadings normalized by their variability (i.e., their standard deviation) estimated using a bootstrap approach (10,000 iterations) and effectively providing the statistical significance (z-score value) of each multivariate load.

**Figure 8:**
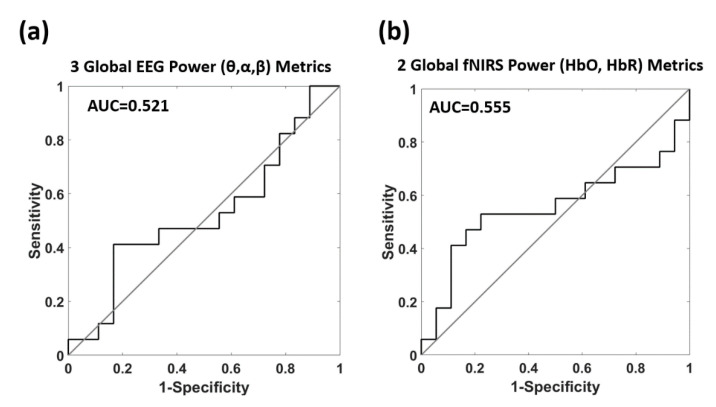
CV classification outcome of the multivariate data-driven analysis predicting AD and HC from the standalone EEG and fNIRS metrics. (**a**) ROC analysis employing the 3 global EEG powers. (**b**) ROC analysis employing the 2 global fNIRS hemoglobin powers.

For comparison with the multivariate data-driven analysis of NC, Figure 8 reports the outcome of the multivariate data-driven analysis trying to predict AD and HC from the standalone EEG and fNIRS metrics. Figure 8a reports the CV outcome of the ROC analysis employing the three EEG global powers, whereas Figure 8b reports the results of the ROC analysis employing the two fNIRS global hemoglobin powers. Using unimodal analysis, a poor AD vs. HC classification was obtained with AUC = 0.521 (z = 0.212, *p* = 0.416) and AUC = 0.555 (z = 0.555, *p* = 0.289) for the EEG and the fNIRS features, respectively. Importantly, the multivariate classification performance based on the multimodal NC evaluation was statistically superior to both the unimodal EEG and fNIRS analysis (AUC = 0.905 vs. 0.521, z = 3.403, *p* = 6.6 × 10^−4^; AUC = 0.905 vs. 0.555, z = 3.115, *p* = 1.8 × 10^−3^).

Notably, when the whole analysis was re-run using half of the total electrodes, eliminating the occipital ones, the difference between AD and HC in the coupling of the theta band and HbO was still statistically significant (t = −2.170, df = 33, *p* = 0.037), whereas the difference in the coupling between the alpha band and HbR vanished (t = −0.530, df = 33, *p* = 0.600), with the multivariate data-driven analysis delivering an AUC of 0.830 (z = 3.329 *p* = 4.4 × 10^−4^).

**Figure 9:**
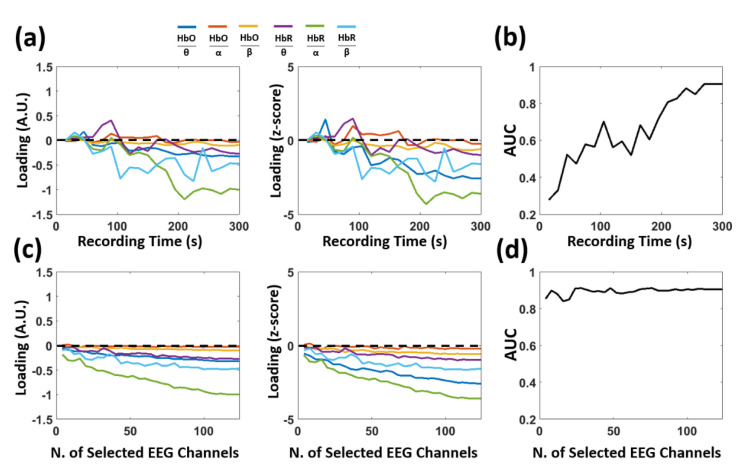
Cross-validated (CV) loadings and classification performance of the multivariate data-driven analysis predicting AD and HC from the 6 NC metrics as a function of either recording time or number of EEG channels employed. (**a**) Left panel: loadings as a function of recording time; right panel: z-scores, obtained as loadings normalized by their standard deviation (estimated using bootstrapping), as a function of recording time. (**b**) Area under the curve (AUC) of the ROC analysis as a function of recording time. (**c**) Left panel: loadings as a function of the number of randomly selected EEG channels; right panel: z-scores, obtained as loadings normalized by their standard deviation, as a function of the number of randomly selected EEG channels. (**d**) AUC of the ROC analysis as a function of the number of randomly selected EEG channels.

Finally, Figure 9 reports the outcome of the multivariate data-driven analysis trying to predict AD and HC from the six NC metrics as a function of either the recording time or the number of randomly selected EEG channels. Figure 9a shows the loadings of the multivariate regression analysis and the associated z-scores as a function of recording time available. Figure 9b instead shows the AUC of the classification outcome, always as a function of the recording time. Indeed, the loadings and the associated z-scores converged to negative values, whereas the AUC increased as a function of recording time. Figure 9c shows the loadings and the associated z-scores as a function of the number of randomly selected EEG channels. Figure 9d shows the AUC of the associated classification outcome. In this case, the loadings quickly assumed negative values, whereas the AUC was quite stable.

## 4. Discussion

### 4.1. Identification of EEG-fNIRS De-Coupling in AD

AD is characterized by a progressive cognitive decline over the course of the disease that slowly disrupts daily life [83]. The exact mechanisms inducing AD symptoms remain largely unknown [84]. Nonetheless, novel neuroimaging modalities and advanced data analysis may enhance the ability to non-invasively assess brain status and subsequently identify its functional deterioration in AD. Such approaches must be suitable for ambulatory settings in order to impact standard clinical practice. To this end, multimodal evaluation of brain activity and NC through synchronous EEG-fNIRS recordings represents a promising tool [46]. EEG and fNIRS are relatively cheap, portable and easy to use technologies. These characteristics make them highly suited for both screening-like and longitudinal ambulatory evaluation of brain diseases. Synchronous EEG and fNIRS recordings may provide relevant information regarding NC, whose deterioration is known to be associated with AD [12,13,14].

In this study, multimodal EEG-fNIRS recordings were performed at rest and they were used to infer NC and evaluate its impairment in AD. The multivariate correlations of whole-head EEG power envelopes in the theta, alpha and beta bands with fNIRS-derived changes in HbO and HbR in frontal and prefrontal cortices were estimated by means of a GLM approach and representative metrics of NC. A significant decrease in correlation, i.e., a global neurovascular un-coupling, was found in AD patients between the theta band power envelope and HbO changes (t = −2.30, df = 33, *p* = 0.028) and between the alpha band power envelope and HbR changes (t = −2.44, df = 33, *p* = 0.020, Figure 5). Hence, for simplicity, we preferred to only report the results about theta and beta bands in the paper as representative of low- and high-frequency EEG band associations with fNIRS hemoglobin oscillations.

The selective un-coupling between specific EEG frequency bands and fNIRS hemoglobin species requires further discussion. The decrease in EEG-fNIRS correlations in AD was found for EEG frequency bands and hemoglobin species where the average NC was significantly above chance in HC (i.e., average β-weight larger than zero, Figure 5). Notably, these results are in line with previous multimodal works combining EEG with BOLD fMRI in HC [45,85].

In HC, a strong negative correlation of EEG alpha power with parietal and frontal cortical activity of BOLD was found [86], whereas a positive correlation was identified at rest between BOLD and theta power of local field potentials in parahippocampal areas [87]. Since the BOLD signal has a coarse negative association with HbR and positive association with HbO [23], both the fMRI findings and the results of this study suggest that the selective de-coupling found in AD between theta and HbO and alpha and HbR could simply reflect a general neurovascular un-coupling that is more pronounced for frequency bands and hemoglobin species where the original physiological association is stronger.

Moreover, none of the six EEG-fNIRS NC metrics were significantly higher in AD with respect to HC. Indeed, these results supported the hypothesis of NC decline in AD. To further highlight the importance of investigating NC in AD with multimodal EEG-fNIRS recordings, unimodal evaluation of either global electrical or hemodynamic brain activity, expressed as average EEG and fNIRS signal powers, did not highlight statistically significant differences between AD and HC. Although not statistically significant, it is interesting to notice that a qualitatively lower EEG power in AD with respect to HC was found for all frequency bands considered coupled with a qualitatively higher fNIRS power for both HbO and HbR. This result may be indicative of a dysregulation of NC which is selectively identified by the multimodal analysis.

### 4.2. Multivariate Exploitation of EEG-fNIRS De-Coupling in AD

To further investigate the NC dysregulation in AD and to exploit the potentialities of the approach presented, a multivariate data-driven analysis was also performed. This analysis concurrently evaluated how the NC metrics (i.e., the standardized regression weights between the three frequency bands and the two hemoglobin species, six features) were modified in AD and it investigated to what extent these NC metrics could be used to identify the pathology. The procedure classified AD and HC relying on the six NC metrics and a multiple linear regression approach. The CV classification outcome was assessed by means of an ROC analysis that delivered AUC = 0.905 (z = 4.090, *p* = 2.17 × 10^−5^, Figure 7a). An interesting result of the multivariate data-driven analysis was that the expected value of the loadings of the linear regression, associated to each of the six NC features was always negative (Figure 7b). This finding further supported the neurovascular un-coupling in AD already found in the univariate analysis. Moreover, the highest statistical relevance of the magnitude of the multivariate loadings was indeed found for the two NCs that were significantly lower in AD based on the univariate analysis (i.e., the link between the theta band and HbO and the link between the alpha band and HbR).

Always in accordance with the univariate analysis, the multivariate data-driven approach used on standalone EEG and fNIRS powers did not provide a classification accuracy of AD and HC above chance (Figure 8). These results further demonstrated the higher sensitivity and specificity to AD of NC metrics evaluated through multimodal approaches with respect to brain activity metrics estimated based on unimodal procedures (AUC = 0.905 vs. 0.521, z = 3.403, *p* = 6.6 × 10^−4^; AUC = 0.905 vs. 0.555, z = 3.115, *p* = 1.8 × 10^−3^). At a specific threshold of the outcome of the multivariate data-driven analysis of NC, a sensitivity of 88.2% and a specificity of 94.5% were obtained, providing additional evidence of the high potentialities of the method.

Of note, whereas the univariate evaluation of the couplings between multiple EEG frequency bands and fNIRS hemoglobin forms might have produced false positives, the multivariate analysis indeed relied on considering all the information at once, greatly decreasing the chance of false positives related to multiple comparison. Indeed, the highly significant multivariate results support the positive findings of the study.

As a further investigation of the findings, the multivariate classification of AD and HC based on EEG-fNIRS NC metrics was evaluated as a function of either the recording time available or the number of EEG channels employed (Figure 9). These are important aspects of the replicability of the results and the implementation of the approach in a clinical environment. With respect to recording time length, both the multivariate loadings and associated z-scores tended to reach stable and negative values after 200 s. The AUC was close to the best performance only when the recording time was close to 300 s, while showing a rather linear decrease in value with shorter recording times. These results corroborated the robustness of the original findings but also highlighted the need for recording durations of several minutes to employ the proposed approach with sufficient sensitivity and specificity. On the contrary, both the multivariate loadings and associated z-scores were stable with the number of randomly selected EEG channels and, importantly, the AUC was very close to the best classification performance even with particularly small numbers of channels used. These findings highlighted the applicability of the approach in a clinical environment where a small number of electrodes is generally available.

### 4.3. Study Limitations

Several limitations of the study must be commented upon and might be addressed in future studies.

Firstly, whereas the high-density EEG provided whole-head coverage, the fNIRS layout allowed us to limit the investigation to the frontal and prefrontal cortices. The layout choice was driven by practical limitations of the available fNIRS instrumentation that provided a limited number of optodes. The choice to cover the frontal and prefrontal cortices was led by the higher sensitivity of the fNIRS optodes in areas not covered by hair and by the involvement of these areas in AD-related cognitive impairment, although to a lower extent with respect to other areas, such as the medial temporal lobe [88,89]. In order to control for the different head coverage levels of EEG and fNIRS, the analysis was run again, but by selectively choosing EEG signals acquired with half of the electrodes all located on the frontal and prefrontal brain areas. With this analysis, the difference between AD and HC in the coupling of the theta band and HbO remained unaltered (t = −2.170, df = 33, *p* = 0.037), whereas the difference in the coupling between the alpha band and HbR still pointed toward a reduction of the linkage strength in AD but vanished statistically (t = −0.530, df = 33, *p* = 0.600). Because of the reduced effect in the coupling between the alpha band and HbR, the multivariate data-driven analysis delivered an AUC of 0.830, which was below the original one of 0.905. These findings suggest that the analysis of the whole-head EEG signal identified an increase in global alpha activity (known to be associated with brain electrical activity de-activation) that induced a decrease in HbR (known to be associated with brain hemodynamic de-activation) in frontal and prefrontal areas and that the alteration of this effect in AD was useful in identifying the patients with the disease. This information was lost when limiting the useful electrodes to those located in the frontal region because of the diminished sensitivity of those electrodes to the alpha band activity. This result, coupled with the findings regarding the classification performance as a function of the number of randomly selected EEG channels, suggested that, although a limited number of electrodes can be used to exploit the proposed approach, such electrodes should be evenly distributed across the scalp. However, further studies should be performed with a whole-head fNIRS system to assess a global NC estimated using whole-brain hemodynamic information in conjunction with electrical activity. Nonetheless, it might be possible that a global NC assessed through whole-head fNIRS may deliver a lower sensitivity for AD. In fact, since the pathology is associated with a cognitive decline often related to frontal cortex activity dysregulation, AD may be characterized by a selective disruption of fNIRS modulation within the frontal lobes in response to global EEG oscillations [90]. Particularly, it could be worth extending the fNIRS investigation to other brain regions highly impaired by AD, such as the medial temporal lobe [89].

Secondly, always with reference to the electrode and optode distribution on the scalp, a less relevant issue, mainly driven by mechanical limitations, was associated with the fact that the EEG electrodes where not placed on the same scalp locations as the optodes nor were exactly in between an optical source and a detector. However, we should further stress that EEG, due to volume conduction effects, is intrinsically a low spatial resolution technology. Indeed, a displacement of EEG sensors of a few centimeters does not drastically modify the signal recorded. In this perspective, a small displacement between fNIRS optodes and EEG electrodes does not affect the assessment of a global NC using EEG-fNIRS, also considering that the global signal was extracted using a high-density EEG system which has an average distance between sensors well below the minimum wavelength (corresponding to the maximum spatial frequency component) of the measured voltages across the head [91]. This statement was indeed heavily supported by the results regarding the stability of the proposed approach as a function of the number of randomly selected EEG channels used.

Thirdly, although the optical probes were located with reference to the EEG 10/20 system, an accurate co-registration of the optical sensors with a specific subject anatomy (e.g., structural MRI of the head) was missing. In fact, also for this reason, only global estimates of NC were evaluated, effectively not accounting for its spatial characteristics. Further studies should indeed be focused on assessing the spatial features of NC modifications in AD. However, although the spatial characteristics of NC may provide additional information, a global metric of NC can be more directly employed in a clinical environment. In fact, providing an accurate spatial localization of electrical and hemodynamic brain activity with EEG and fNIRS requires several methodological steps that make such an approach impractical for employment outside research environments (e.g., co-registration with an anatomical image of the subject, implementation of forward and inverse approaches [92,93]).

Fourthly, although the multivariate data-driven analysis delivered a high classification performance, further studies should be conducted to increase the overall sample size of the population. In fact, these approaches rely on analyses that might drastically increase their performances when large sample sizes are available [80]. When a larger data sample is available, the analysis of NC might be extended beyond the six global metrics that were used in this work, for example, considering more than one principal component for each EEG frequency band, and with the possibilities to explore non-linearities in the data.

Fifthly, in vivo neuroimaging metrics, such as the NC measured with multimodal EEG-fNIRS recordings, are certainly not suitable to understand the underlying microscopic mechanisms associated with the macroscopic manifestations. The drop in correlation between EEG and fNIRS signals identified in AD patients was plausibly associated with a disruption of signal pathways in astrocytes, vascular smooth muscle cells and endothelia, causing a decreased or slower cerebrovascular reactivity in NC, but further study should be performed to fully explain the findings [15].

Finally, this study was limited to the comparison of NC between AD and HC. In order to employ this approach for diagnostic purposes, the capabilities of this method to identify AD and to differentiate it from other similar pathologies should be assessed, effectively providing a procedure able to perform a differential diagnosis [94]. This is particularly relevant since other forms of dementia might indeed exhibit similar neurovascular un-coupling to that found in AD. In fact, since AD shares similar symptomologies with other neurological pathologies, early diagnosis and monitoring of AD represents a major issue for clinicians.

### 4.4. Study Significance

The findings clearly demonstrated a neurovascular de-coupling in AD and highlighted how this de-coupling might be exploited to identify AD at an early stage of the disease. Importantly, the approach implemented can be easily translated to clinical, ambulatory settings, thanks to the portability and usability of both EEG and fNIRS. Thanks to the extraction of global metrics of brain activity to assess NC, this procedure does not require EEG and fNIRS sensor co-registration to an anatomical image, which would imply the usage of expensive and time-consuming imaging techniques (e.g., MRI or CT). The unbiased cross-validated framework built to assess the performance of the multivariate approach in identifying AD through global EEG-fNIRS NC clearly suggests the reliability of the proposed method, despite the small number of subjects included in the study.

### 4.5. Future Perspectives

The neuroimaging approaches presented in this work, unifying novel ecological multimodal resting-state recordings of brain activity and multivariate data-driven analysis of the signals acquired, could support early AD diagnosis, potentially improving treatment efficacy and decreasing societal economic effort. In fact, the actual measurement does not require specifically trained personnel, and the algorithm, once implemented, can be easily used by clinicians.

## 5. Conclusions

In this study, NC was evaluated in AD and HC by means of multimodal EEG-fNIRS recordings performed at rest. Six metrics of global NC of theta, alpha and beta band power envelopes with HbO and HbR changes were extracted by using whole-head high-density EEG recordings, frontal and prefrontal cortex fNIRS measures and a GLM approach. Univariate analysis of the six NC features showed a statistically significant decrease in the coupling between theta and HbO and between alpha and HbR. Importantly, no significant effects were found when analyzing the average power of the signals of standalone modalities. A linear, multivariate and data-driven approach to the classification of AD and HC from the six NC metrics delivered a high classification performance with AUC = 0.905. Moreover, the procedure provided negative loadings for all the features, suggesting a general neurovascular un-coupling in AD. Although such an approach should be tested by taking into account other similar pathologies that can affect NC in order to provide a differential diagnosis, the findings indeed demonstrated the capabilities of resting-state EEG-fNIRS to assess NC in ecological ambulatory settings and corroborated the hypothesis of NC disruption in AD, paving the way to the employment of such neuroimaging approaches to support early AD diagnosis.

## Figures and Tables

**Table 1:** Demographic information of Alzheimer’s disease (AD) and healthy controls (HC) involved in the study.

	AD	HC	*p*-Value
Gender (M–F)	9–8	9–9	/
Age (years) (mean ± SD)	75.1 ± 7.1	71.4 ± 7.8	0.1354
Education (years) (mean ± SD)	9.6 ± 4.5	10.9 ± 4.3	0.3719
Mini Mental State Examination (MMSE) score (mean ± SD)	22.9 ± 3.2	27.5 ± 2.2	4.05 × 10^−6^

**Table 2:** Confusion matrix obtained by selecting a specific threshold of the output of the multivariate linear regression classifying AD and HC from the 6 NC metrics.

	Label	HC	AD	TOT
**Counts**	HC	17	1	18
	AD	2	15	17
**%**	HC	94.5%	5.5%	100%
	AD	11.8%	88.2%	100%

## Data Availability

The data presented in this study are available on request from the corresponding author. The data are not publicly available due to privacy issues.

## Abbreviations

**Abbreviation**	**Meaning**
AD	Alzheimer’s Disease
AUC	Area Under the Curve
BOLD	Blood Oxygen Level-Dependent
CBF	Cerebral Blood Flow
CMRO_2_	Cerebral Metabolic Rate of Oxygen
CV	Cross-Validation
CVR	Cerebrovascular Reactivity
DPF	Differential Pathlength Factor
EEG	Electroencephalography
fMRI	functional Magnetic Resonance Imaging
fNIRS	functional Near-Infrared Spectroscopy
GLM	General Linear Model
HbO	Oxy-hemoglobin
HbR	Deoxy-hemoglobin
HC	Healthy Controls
HRF	Hemodynamic Response Function
ICA	Independent Component Analysis
MMSE	Mini Mental State Examination
NC	Neurovascular Coupling
OEF	Oxygen Extraction Fraction
PCA	Principal Component Analysis
PMT	Photomultiplier Tube
ROC	Receiver Operating Characteristic

## References

[1] Dubois B., Feldman H.H., Jacova C., DeKosky S.T., Barberger-Gateau P., Cummings J., Delacourte A., Galasko D., Gauthier S., Jicha G. (2007). Research criteria for the diagnosis of alzheimer’s disease: Revising the NINCDS–ADRDA criteria. Lancet Neurol..

[2] Perpetuini D., Cardone D., Bucco R., Zito M., Merla A. (2018). Assessment of the autonomic response in alzehimer’s patients during the execution of memory tasks: A functional thermal imaging study. Curr. Alzheimer Res..

[3] Filippini C., Perpetuini D., Cardone D., Chiarelli A.M., Merla A. (2020). Thermal Infrared Imaging and Artificial Intelligence Techniques Can Support Mild Alzheimer Disease Diagnosis.

[4] Reitz C., Brayne C., Mayeux R. (2011). Epidemiology of alzheimer disease. Nat. Rev. Neurol..

[5] Finch C.E., Cohen D.M. (1997). Aging, metabolism, and alzheimer disease: Review and hypotheses. Exp. Neurol..

[6] Alsunusi S., Kumosani T.A., Glabe C.G., Huwait E.A., Moselhy S.S. (2020). In Vitro study of the mechanism of intraneuronal β-amyloid aggregation in alzheimer’s disease. Arch. Physiol. Biochem..

[7] Braak H., Braak E. (1991). Neuropathological stageing of alzheimer-related changes. Acta Neuropathol..

[8] Mamun A.A., Uddin M.S., Mathew B., Ashraf G.M. (2020). Toxic tau: Structural origins of tau aggregation in alzheimer’s disease. Neural Regen Res.

[9] Mandelkow E.-M., Mandelkow E. (1998). Tau in alzheimer’s disease. Trends Cell Biol..

[10] Gómez-Isla T., Hollister R., West H., Mui S., Growdon J.H., Petersen R.C., Parisi J.E., Hyman B.T. (1997). Neuronal loss correlates with but exceeds neurofibrillary tangles in alzheimer’s disease. Ann. Neurol..

[11] Koper M.J., Van Schoor E., Ospitalieri S., Vandenberghe R., Vandenbulcke M., von Arnim C.A.F., Tousseyn T., Balusu S., De Strooper B., Thal D.R. (2020). Necrosome complex detected in granulovacuolar degeneration is associated with neuronal loss in alzheimer’s disease. Acta Neuropathol..

[12] Grammas P. (2011). Neurovascular dysfunction, inflammation and endothelial activation: Implications for the pathogenesis of alzheimer’s disease. J. Neuroinflamm..

[13] Nelson A.R., Sweeney M.D., Sagare A.P., Zlokovic B.V. (2016). Neurovascular dysfunction and neurodegeneration in dementia and alzheimer’s disease. Biochim. Et Biophys. Acta (Bba) Mol. Basis Dis..

[14] Zlokovic B.V. (2005). Neurovascular mechanisms of alzheimer’s neurodegeneration. Trends Neurosci..

[15] Kisler K., Nelson A.R., Montagne A., Zlokovic B.V. (2017). Cerebral blood flow regulation and neurovascular dysfunction in alzheimer disease. Nat. Rev. Neurosci..

[16] Kotliar K., Hauser C., Ortner M., Muggenthaler C., Diehl-Schmid J., Angermann S., Hapfelmeier A., Schmaderer C., Grimmer T. (2017). Altered neurovascular coupling as measured by optical imaging: A biomarker for alzheimer’s disease. Sci. Rep..

[17] Perpetuini D., Chiarelli A.M., Cardone D., Filippini C., Bucco R., Zito M., Merla A. (2019). Complexity of frontal cortex FNIRS Can support alzheimer disease diagnosis in memory and visuo-spatial tests. Entropy.

[18] Chow B.W., Nuñez V., Kaplan L., Granger A.J., Bistrong K., Zucker H.L., Kumar P., Sabatini B.L., Gu C. (2020). Caveolae in CNS arterioles mediate neurovascular coupling. Nature.

[19] Croce P., Zappasodi F., Merla A., Chiarelli A.M. (2017). Exploiting neurovascular coupling: A bayesian sequential monte carlo approach applied to simulated EEG FNIRS data. J. Neural Eng..

[20] Croce P., Basti A., Marzetti L., Zappasodi F., Del Gratta C. (2016). EEG? FMRI bayesian framework for neural activity estimation: A simulation study. J. Neural Eng..

[21] Haydon P.G., Carmignoto G. (2006). Astrocyte control of synaptic transmission and neurovascular coupling. Physiol. Rev..

[22] Uludağ K., Dubowitz D.J., Yoder E.J., Restom K., Liu T.T., Buxton R.B. (2004). Coupling of cerebral blood flow and oxygen consumption during physiological activation and deactivation measured with FMRI. NeuroImage.

[23] Buxton R.B. (2009). Introduction to Functional Magnetic Resonance Imaging: Principles and Techniques.

[24] Penny W.D., Friston K.J., Ashburner J.T., Kiebel S.J., Nichols T.E. (2011). Statistical Parametric Mapping: The Analysis of Functional Brain Images.

[25] Parker A., Derrington A., Blakemore C., Logothetis N.K. (2002). The neural basis of the blood–oxygen–level–dependent functional magnetic resonance imaging signal. Philos. Trans. R. Soc. Lond. Ser. B Biol. Sci..

[26] Ferrari M., Quaresima V. (2012). A brief review on the history of human functional near-infrared spectroscopy (FNIRS) development and fields of application. Neuroimage.

[27] Quaresima V., Ferrari M. (2019). Functional Near-infrared spectroscopy (FNIRS) for assessing cerebral cortex function during human behavior in natural/social situations: A concise review. Organ. Res. Methods.

[28] Arenth P.M., Ricker J.H., Schultheis M.T. (2007). Applications of functional near-infrared spectroscopy (FNIRS) to Neurorehabilitation of cognitive disabilities. Clin. Neuropsychol..

[29] Costantini M., Vacri A.D., Chiarelli A.M., Ferri F., Romani G.L., Merla A. (2013). Studying social cognition using near-infrared spectroscopy: The case of social simon effect. JBO JBOPFO.

[30] Irani F., Platek S.M., Bunce S., Ruocco A.C., Chute D. (2007). Functional near infrared spectroscopy (FNIRS): An emerging neuroimaging technology with important applications for the study of brain disorders. Clin. Neuropsychol..

[31] Naseer N., Hong K.-S. (2015). FNIRS-based Brain-computer interfaces: A review. Front. Hum. Neurosci..

[32] Perpetuini D., Bucco R., Zito M., Merla A. (2018). Study of memory deficit in alzheimer’s disease by means of complexity analysis of FNIRS signal. Neurophotonics.

[33] Pinti P., Aichelburg C., Lind F., Power S., Swingler E., Merla A., Hamilton A., Gilbert S., Burgess P., Tachtsidis I. (2015). Using fiberless, wearable FNIRS to monitor brain activity in real-world cognitive tasks. J. Vis. Exp..

[34] Watanabe H., Shitara Y., Aoki Y., Inoue T., Tsuchida S., Takahashi N., Taga G. (2017). Hemoglobin phase of oxygenation and deoxygenation in early brain development measured using FNIRS. Proc. Natl. Acad. Sci. USA.

[35] Bennett C.M., Miller M.B. (2013). FMRI reliability: Influences of Task and experimental design. Cogn. Affect. Behav. Neurosci..

[36] Lindquist M.A., Meng Loh J., Atlas L.Y., Wager T.D. (2009). Modeling the hemodynamic response function in FMRI: Efficiency, bias and mis-modeling. NeuroImage.

[37] Friston K.J., Mechelli A., Turner R., Price C.J. (2000). Nonlinear responses in FMRI: The balloon model, volterra kernels, and other hemodynamics. Neuroimage.

[38] Jasdzewski G., Strangman G., Wagner J., Kwong K.K., Poldrack R.A., Boas D.A. (2003). Differences in the hemodynamic response to event-related motor and visual paradigms as measured by near-infrared spectroscopy. NeuroImage.

[39] Steffener J., Tabert M., Reuben A., Stern Y. (2010). Investigating hemodynamic response variability at the group level using basis functions. NeuroImage.

[40] Niedermeyer E., da Silva F.H.L. (2005). Electroencephalography: Basic Principles, Clinical Applications, and Related Fields.

[41] Hämäläinen M., Hari R., Ilmoniemi R.J., Knuutila J., Lounasmaa O.V. (1993). Magnetoencephalography—Theory, instrumentation, and applications to noninvasive studies of the working human brain. Rev. Mod. Phys..

[42] Ritter P., Villringer A. (2006). Simultaneous EEG–FMRI. Neurosci. Biobehav. Rev..

[43] Schulz M., Chau W., Graham S.J., McIntosh A.R., Ross B., Ishii R., Pantev C. (2004). An integrative MEG–FMRI study of the primary somatosensory cortex using cross-modal correspondence analysis. NeuroImage.

[44] Stickland R., Allen M., Magazzini L., Singh K.D., Wise R.G., Tomassini V. (2019). Neurovascular coupling during visual stimulation in multiple sclerosis: A MEG-FMRI STUDY. Neuroscience.

[45] Brueggen K., Fiala C., Berger C., Ochmann S., Babiloni C., Teipel S.J. (2017). Early changes in alpha band power and dmn bold activity in alzheimer’s disease: A Simultaneous resting state EEG-FMRI study. Front. Aging Neurosci..

[46] Chiarelli A.M., Zappasodi F., Pompeo F.D., Merla A. (2017). Simultaneous functional near-infrared spectroscopy and electroencephalography for monitoring of human brain activity and oxygenation: A review. NphNeurow.

[47] Li R., Potter T., Huang W., Zhang Y. (2017). Enhancing Performance of a hybrid EEG-FNIRS system using channel selection and early temporal features. Front. Hum. Neurosci..

[48] Perpetuini D., Chiarelli A.M., Filippini C., Cardone D., Croce P., Rotunno L., Anzoletti N., Zito M., Zappasodi F., Merla A. (2020). Working memory decline in alzheimer’s disease is detected by complexity analysis of multimodal EEG-FNIRS. Entropy.

[49] Davidson R.J., Jackson D.C., Larson C.L. (2000). Human electroencephalography. Handbook of Psychophysiology.

[50] Kiloh L.G., McComas A.J., Osselton J.W. (2013). Clinical Electroencephalography.

[51] Li R., Nguyen T., Potter T., Zhang Y. (2019). Dynamic cortical connectivity alterations associated with alzheimer’s disease: An EEG and FNIRS integration study. Neuroimage Clin..

[52] Cicalese P.A., Li R., Ahmadi M.B., Wang C., Francis J.T., Selvaraj S., Schulz P.E., Zhang Y. (2020). An EEG-FNIRS hybridization technique in the four-class classification of alzheimer’s disease. J. Neurosci. Methods.

[53] Dutta A., Jacob A., Chowdhury S.R., Das A., Nitsche M.A. (2015). EEG-NIRS based assessment of neurovascular coupling during anodal transcranial direct current stimulation—A stroke case series. J. Med. Syst..

[54] Hendrikx D., Smits A., Lavanga M., De Wel O., Thewissen L., Jansen K., Caicedo A., Van Huffel S., Naulaers G. (2019). Measurement of neurovascular coupling in neonates. Front. Physiol..

[55] Othman M.H., Bhattacharya M., Møller K., Kjeldsen S., Grand J., Kjaergaard J., Dutta A., Kondziella D. (2020). Resting-state NIRS–EEG in unresponsive patients with acute brain injury: A proof-of-concept study. Neurocrit Care.

[56] Csipo T., Mukli P., Lipecz A., Tarantini S., Bahadli D., Abdulhussein O., Owens C., Kiss T., Balasubramanian P., Nyúl-Tóth Á. (2019). Assessment of Age-related decline of neurovascular coupling responses by functional near-infrared spectroscopy (FNIRS) in humans. GeroScience.

[57] Chiarelli A.M., Romani G.L., Merla A. (2014). Fast optical signals in the sensorimotor cortex: General linear convolution model applied to multiple source–detector distance-based data. NeuroImage.

[58] Monti M.M. (2011). Statistical analysis of FMRI time-series: A critical review of the GLM approach. Front. Hum. Neurosci..

[59] Perpetuini D., Cardone D., Filippini C., Chiarelli A.M., Merla A. (2019). Modelling impulse response function of functional infrared imaging for general linear model analysis of autonomic activity. Sensors.

[60] Li R., Zhao C., Wang C., Wang J., Zhang Y. (2020). Enhancing FNIRS analysis using EEG rhythmic signatures: An EEG-informed FNIRS analysis study. IEEE Trans. Biomed. Eng..

[61] Folstein M.F., Folstein S.E., McHugh P.R. (1975). “Mini-Mental State”: A practical method for grading the cognitive state of patients for the clinician. J. Psychiatr. Res..

[62] Tucker D.M. (1993). Spatial sampling of head electrical fields: The geodesic sensor net. Electroencephalogr. Clin. Neurophysiol..

[63] Homan R.W., Herman J., Purdy P. (1987). Cerebral location of international 10–20 system electrode placement. Electroencephalogr. Clin. Neurophysiol..

[64] Brigadoi S., Cooper R.J. (2015). How short is short? Optimum source-detector distance for short-separation channels in functional near-infrared spectroscopy. Neurophotonics.

[65] Tachtsidis I., Scholkmann F. (2016). False positives and false negatives in functional near-infrared spectroscopy: Issues, challenges, and the way forward. NPh.

[66] Gagnon L., Cooper R.J., Yücel M.A., Perdue K.L., Greve D.N., Boas D.A. (2012). Short separation channel location impacts the performance of short channel regression in NIRS. NeuroImage.

[67] Dehghani H., Eames Matthew E., Yalavarthy Phaneendra K., Davis Scott C., Srinivasan S., Carpenter Colin M., Pogue Brian W., Paulsen Keith D. (2008). Near infrared optical tomography using NIRFAST: Algorithm for numerical model and image reconstruction. Commun. Numer. Methods Eng..

[68] Barbati G., Porcaro C., Zappasodi F., Rossini P.M., Tecchio F. (2004). Optimization of an independent component analysis approach for artifact identification and removal in magnetoencephalographic signals. Clin. Neurophysiol..

[69] Croce P., Zappasodi F., Marzetti L., Merla A., Pizzela V., Chiarelli A.M. (2018). Deep convolutional neural networks for feature-less automatic classification of independent components in multi-channel electrophysiological brain recordings. IEEE Trans. Biomed. Eng..

[70] Chiarelli A.M., Maclin E.L., Fabiani M., Gratton G. (2015). A Kurtosis-based wavelet algorithm for motion artifact correction of FNIRS data. NeuroImage.

[71] Kocsis L., Herman P., Eke A. (2006). The modified beer–lambert law revisited. Phys. Med. Biol..

[72] Zijlstra W.G., Buursma A., Meeuwsen-van der Roest W.P. (1991). Absorption spectra of human fetal and adult oxyhemoglobin, de-oxyhemoglobin, carboxyhemoglobin, and methemoglobin. Clin. Chem..

[73] Chiarelli A.M., Perpetuini D., Filippini C., Cardone D., Merla A. (2019). Differential pathlength factor in continuous wave functional near-infrared spectroscopy: Reducing hemoglobin’s cross talk in high-density recordings. NPh.

[74] Scholkmann F., Wolf M. (2013). General equation for the differential pathlength factor of the frontal human head depending on wavelength and age. J. Biomed. Opt..

[75] Musso F., Brinkmeyer J., Mobascher A., Warbrick T., Winterer G. (2010). Spontaneous brain activity and EEG microstates. A novel EEG/FMRI analysis approach to explore resting-state networks. Neuroimage.

[76] Yuan H., Zotev V., Phillips R., Drevets W.C., Bodurka J. (2012). Spatiotemporal dynamics of the brain at rest—Exploring EEG microstates as electrophysiological signatures of BOLD resting state networks. NeuroImage.

[77] Hogg R., McKean J., Craig A. (2005). Introduction to Mathematical Statistics.

[78] Johnson R.A., Wichern D.W. (2006). Multivariate analysis. Encyclopedia of Statistical Sciences.

[79] Hastie T., Tibshirani R., Friedman J. (2009). The Elements of Statistical Learning: Data Mining, Inference, and Prediction.

[80] Liu R., Gillies D.F. (2016). Overfitting in linear feature extraction for classification of high-dimensional image data. Pattern Recognit..

[81] Kohavi R. (1995). A study of cross-validation and bootstrap for accuracy estimation and model selection. Proceedings of the 14th International Joint Conference on Artificial Intelligence—Volume 2.

[82] Esterman M., Tamber-Rosenau B.J., Chiu Y.-C., Yantis S. (2010). Avoiding non-independence in FMRI data analysis: Leave one subject out. NeuroImage.

[83] Petersen R.C. (2000). Mild cognitive impairment: Transition between aging and alzheimer’s disease. NEUROLOGIA.

[84] Serrano-Pozo A., Frosch M.P., Masliah E., Hyman B.T. (2011). Neuropathological alterations in alzheimer disease. Cold Spring Harb. Perspect. Med..

[85] Logothetis N.K., Pauls J., Augath M., Trinath T., Oeltermann A. (2001). Neurophysiological investigation of the basis of the FMRI signal. Nature.

[86] Laufs H., Kleinschmidt A., Beyerle A., Eger E., Salek-Haddadi A., Preibisch C., Krakow K. (2003). EEG-correlated FMRI of human alpha activity. NeuroImage.

[87] Ekstrom A., Suthana N., Millett D., Fried I., Bookheimer S. (2009). Correlation between BOLD FMRI and theta-band local field potentials in the human hippocampal area. J. Neurophysiol..

[88] Salat D.H., Kaye J.A., Janowsky J.S. (2001). Selective preservation and degeneration within the prefrontal cortex in aging and alzheimer disease. Arch. Neurol..

[89] Brun A., Gustafson L. (1976). Distribution of cerebral degeneration in alzheimer’s disease. Arch. Für Psychiatr. Und Nervenkrankh..

[90] Mahady L., Nadeem M., Malek-Ahmadi M., Chen K., Perez S.E., Mufson E.J. (2018). Frontal cortex epigenetic dysregulation during the progression of alzheimer’s disease. J. Alzheimer’s Dis..

[91] Buzsáki G., Anastassiou C.A., Koch C. (2012). The origin of extracellular fields and currents—EEG, ECoG, LFP and spikes. Nat. Rev. Neurosci..

[92] Chiarelli A.M., Maclin E.L., Low K.A., Mathewson K.E., Fabiani M., Gratton G. (2016). Combining energy and laplacian regularization to accurately retrieve the depth of brain activity of diffuse optical tomographic data. J. Biomed. Opt..

[93] Chiarelli A.M., Maclin E.L., Low K.A., Fabiani M., Gratton G. (2015). Comparison of procedures for co-registering scalp-recording locations to anatomical magnetic resonance images. J. Biomed. Opt..

[94] Swainson R., Hodges J.R., Galton C.J., Semple J., Michael A., Dunn B.D., Iddon J.L., Robbins T.W., Sahakian B.J. (2001). Early detection and differential diagnosis of alzheimer’s disease and depression with neuropsychological tasks. DEM.

